# Association of Age with the Expression of Hypoxia-Inducible Factors HIF-1α, HIF-2α, HIF-3α and VEGF in Lung and Heart of Tibetan Sheep

**DOI:** 10.3390/ani9090673

**Published:** 2019-09-11

**Authors:** Yanyu He, John S Munday, Matthew Perrott, Guan Wang, Xiu Liu

**Affiliations:** 1 College of Animal Science and Technology/Gansu Provincial Key Laboratory of Ariland Crop Science, Gansu Agricultural University, Lanzhou 730070, China; heyanyuxy@sina.com; 2 School of Veterinary Science, Massey University, Palmerston North 4410, New Zealand; m.r.perrott@massey.ac.nz; 3 College of Animal Science and Technology/Gansu Key Laboratory of Herbivorous Animal Biotechnology, Gansu Agricultural University, Lanzhou 730070, China; 13680740a@sina.com

**Keywords:** hypoxia, HIFs, VEGF, heart and lung, macrophage, Tibetan sheep

## Abstract

**Simple Summary:**

The heart and lung play an essential role in physiological homeostasis, especially in a hypoxic environment. The effect of aging on HIF-1α, HIF-2α, HIF-3α and VEGF expression in the heart and lung of Tibetan sheep that were adapted to hypoxia was evaluated in this study. We conclude that HIF-3a and VEGF are important in how the heart responds to hypoxia and that HIF-1a and HIF-2a may help mediate the adaptation by the sheep to hypoxia. The results suggested that the altered expression of these proteins due to hypoxia is regulated at the protein as well as gene levels. The expression of these proteins in alveolar macrophages suggests these cells play an important role in adaption to hypoxia. The research could provide insight into the role of inflammation in response to reduced alveolar PO2, and is useful in understanding how age influences the hypoxia adaption mechanisms of the heart and lung. This may allow a better understanding of chronic mountain sickness that is commonly observed in Tibetan people living at high altitude on the Qinghai-Tibetan plateau.

**Abstract:**

Hypoxia-inducible factors (HIFs) play an important role in mediating the physiological response to low oxygen environments. However, whether the expression of HIFs changes with age is unknown. In the present study, the effect of aging on HIF-1α, HIF-2α, HIF-3α and VEGF expression in the heart and lung of 30 Tibetan sheep that were adapted to hypoxia was evaluated. The 30 sheep were subdivided into groups of 10 animals that were 1, 2 or 6 years of age. Immunohistochemistry for HIF-1α, HIF-2α, HIF-3α and VEGF revealed that the immunostaining intensity of VEGF protein in the heart and lung was significantly higher than the intensity of immunostaining against the HIFs (*p* < 0.05). HIF-1α and HIF-2α protein translocated into the nucleus of cardiac muscle cells. However, immunostaining for HIF-3α was restricted to the cytoplasm of the myocardial cells. Immunostaining for HIF-1α, HIF-2α, HIF-3α and VEGF was detected within alveolar macrophages. The concentration of HIF-1α and HIF-2α was higher in the lung of 1-year-old than 6-year-old sheep (*p* < 0.05). In contrast, HIF-3α and VEGF immunostaining was most prominent in the hearts of the oldest sheep. However, when RT-PCR was used to evaluate RNA within the tissues, the expression of all four studied genes was higher in the lung than in the heart in the 1-year-old sheep (*p* < 0.05). Furthermore, VEGF and HIF-3α gene expression was higher in the heart from 1-year old than 6-year old sheep (*p* < 0.05). However, in the lung, HIF-1α and HIF-2α gene expression was lower in 1-year old than 6-year old sheep (*p* < 0.05). We conclude that HIF-3α and VEGF may play be important in how the heart responds to hypoxia. Additionally, HIF-1α and HIF-2α may have a role in the adaptation of the lung to hypoxia. The expression of these proteins in alveolar macrophages suggests a potential role of these cells in the physiological response to hypoxia. These results are useful in understanding how age influences the hypoxia adaption mechanisms of the heart and lung and may help to better understand chronic mountain sickness that is commonly observed in Tibetan people living on the Qinghai-Tibetan plateau.

## 1. Introduction

The Tibetan highlands are, on average, 4000 m about sea level and are inhabited by both humans and animals [1,2]. To survive and thrive raises challenges to mammalian physiology. However, some local animal species such as the Yak, Tibetan goat, Tibetan sheep, Tibetan chicken and Tibetan pig appear tolerant to the hypoxia that results from the high altitude [3,4]. Undoubtedly, changes in the heart and lung are the key to the high-altitude hypoxia adaptation. Raising these high-altitude adapted animals is an essential part of life for many Tibetan people so that maximizing production is economically important.

Animals that are not adapted to the hypoxia that occurs at high altitude can develop ‘high-mountain’ (also known as ‘brisket’) disease that is characterized by pulmonary hypertension and right ventricular hypertrophy. Chronic mountain sickness is also an important disease of people of the highlands [5].

Tibetan sheep are mainly distributed on the Qinghai-Tibet Plateau in the highland and typically live at an altitude of 3000–5000 m. This farmed species (*Aries ammon*) were derived from wild sheep and are remarkably tolerant to the hypoxia of high altitudes. Tibetan sheep are one of the main sources of economic activity to local farmers and herdsmen with numbers estimated to be around 23 million in 2008 [6].

Hypoxia inducible factors (HIFs), including HIF-1α, HIF-2α and HIF-3α, are crucial regulators for maintaining biological homeostasis under conditions of low oxygen and play a role in the remodeling of tissue during physiological adaption to moderate levels of systemic hypoxia [7,8,9]. Hypoxia is also one of the potent stimulators for vascular endothelial growth factor (VEGF) expression [10]. Following hypoxia, HIF-1α is stabilized and is targeted to the nucleus where it binds to the hypoxia response elements in the 5′ flanking region of the VEGF gene. VEGF expression then plays an important role in the vascular changes described during chronic hypoxia. Previous research in yaks revealed that the amount of HIF-1α in the myocardium was higher in 2-year old animals than in younger and older yaks. However, the presence of VEGF in the myocardium increased throughout the life of the animal with the oldest yaks having the highest concentrations of myocardial VEGF [11,12,13]. In contrast to yak, there are relatively few studies evaluating the response of Tibetan sheep to hypoxia and the changes in the adaption process relative to age have not previously been evaluated in this species. In addition, there has been little previous research examining HIF-2α and HIF-3α in hypoxic animals. Therefore, the aim of the present study was to examine the expression pattern of HIF-1α, HIF-2α, HIF-3α and VEGF in the lung and heart of Tibetan sheep and the compare the expression patterns in sheep of three different ages. The detection of significant age-related changes would help to understand how the response to hypoxia changes with age and could provide important information regarding ways to prevent hypoxia-induced disease both in domestic animals and in people.

## 2. Materials and Methods

### 2.1. Tibetan Sheep

A total of 30 Tibetan sheep were purchased from a farm in Luqu County (altitude 4500 m) of Gansu Province (China). Three groups of 10 sheep, were included in this study and their age status (1, 2 or 6 years) were confirmed. These three age groups were selected to represent sheep that were older lambs, young adults, and mature adults. As rates of chronic mountain sickness in people increase rapidly from 30 years of age, the mature adult sheep in this study were included to represent this age group in people. All animals were sampled after slaughter at a slaughterhouse that complied with local regulations.

### 2.2. Immunohistochemistry Detection of HIF-1α, HIF-2α, HIF-3α and VEGF

The same area of the heart (left and right ventricle wall) and lung (left and right lung lobe) were taken from each sheep and fixed in 4% formalin in PBS at 4 °C for 24 h. The fixed samples were dehydrated, embedded with paraffin, and cut into 5-μm-thick sections. The sections were deparaffinized with xylene and ethanol. Briefly, the endogenous peroxidase was blocked with 1.2% hydrogen peroxide in absolute methanol, followed by antigen retrieval in 10 mM citrate buffer (pH 6.0) for 30 min in a microwave oven. After washing in PBS, the sections were preincubated for 60 min at room temperature in 5% horse serum to avoid non-specific immunostaining. Between the separate steps, the sections were rinsed with cold PBS/Triton X-100. Subsequently, they were incubated in a humid chamber overnight at 4 °C with a primary antibody of rabbit anti-HIF-1α, anti-HIF-2α, anti-HIF-3α or anti-VEGF polyclonal antibody (all antibodies diluted 1:200, Bioss, China, bs-20398R, bs-1447R, bs-5989R and bs-1665R), as described previously [13]. It is acknowledged that the specificity of these four anti-rabbit antibodies for the sheep proteins is unknown. The sections were then incubated with the anti-rabbit secondary antibody labelled with horse radish peroxidase (HRP). Immunostaining was detected using 3–3′-diaminobenzidine and sections were counterstained with haematoxylin. A no antibody negative control was also included. Positive immunostaining was brown within the sections.

To quantify the integrated optical density (IOD) of immunoreactivity of sections of heart and lung, images of immunostaining sections were taken on an automated Olympic DP70 microscope and the IOD (Integrated optical density) measured using Image Pro-plus 6.0 software (Media Cybernetics, Inc: Bethesda, MD, USA). The IOD value (also known as integrated absorbance), is the sum of the optical density values of each pixel within the measured structure. To measure the IOD, 100 microscopic fields were analyzed from the heart and lung from each animal included in the study.

### 2.3. Detection of HIF-1α, HIF-2α, HIF-3α and VEG by ELISA

HIF-1α, HIF-2α, HIF-3α and VEGF protein concentration in heart and lung were directly measured by ELISA. To measure this, 100 mg of fresh tissue was triturated using a homogenizer and mixed with 500 μL PBS. The solution was then centrifuged for 10 min at 3000 g at 4 °C and the supernatant collected. The protein was measured and a solution containing 100 μg protein was analyzed according to the manufacturer’s instructions (VEGF and HIFs ELISA Kit, Bio-Swamp, Wuhan Beinglay Biotech Co., LTD, Wuhan, China). The PBS solution used for grinding tissue was analyzed as a control. The absorbance was measured at 450 nm. The concentrations of HIF-1α, HIF-2α, HIF-3α and VEGF were calculated from corresponding with the HIF-1α, HIF-2α, HIF-3α and VEGF standard curves.

### 2.4. RT-PCR

Total RNA was extracted from un-fixed heart and lung samples with Trizol reagent (Invitrogen, Carlsbad, CA, USA). First-strand cDNA was synthesized from 1 μg of purified total RNA, using the PrimeScriptTM RT kit (Takara, Otsu, Japan) according to the manufacturer’s instructions. SYBR^®^ Premix Ex TaqTM II (Takara, Otsu, Japan) was used for measuring the relative expression level of hypoxia-associated genes in various tissues by real-time quantitative PCR (RT-qPCR) using a CFX96 Real-Time PCR Detection System (BioRad, Hercules, CA, USA). Glyceraldehyde-3-phosphate dehydrogenase (GAPDH) and β-actin were used as endogenous references for normalization of targeted mRNA profiles. The primers for RT-qPCR of HIF1α, HIF2α, HIF3α, VEGF and the reference gene 18S were designed by DNASTAR software (Table S1), which were verified by general PCR, and the melting curves of every genes are only single peaks (Figure S1). The results showed that the fluorescence quantitative PCR amplification products are specific, and the relative quantitative expression of HIF1α, HIF2α, HIF3α, and VEGF genes can be performed. The variance of gene expression was analyzed by SPSS software.

### 2.5. Measurement and Statistical Analysis

Data were expressed as the mean ± SD and were analysed by one-way ANOVA using SPSS software. Tukey’s honestly significant difference (HSD) post hoc test was used to investigate differences between gene expression and protein concentrations as measured by ELISA. A *p* value of < 0.05 was considered statistically significant.

## 3. Results

### 3.1. Immunostaining for HIF-1α, HIF-2α, HIF-3α and VEGF in the Heart and Lung of Tibetan Sheep

**Figure 1:**
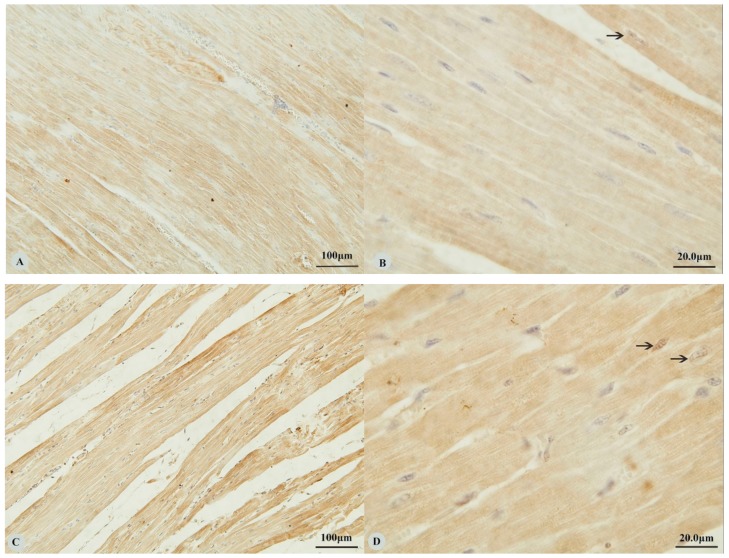

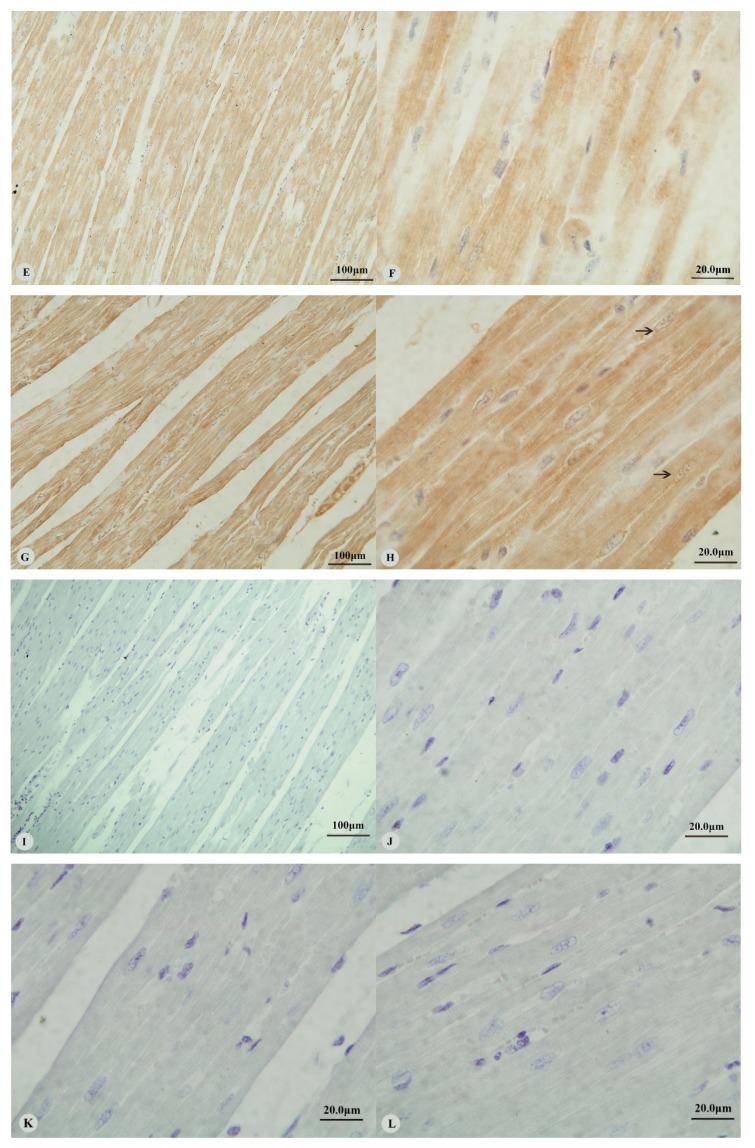
Immunostaining of four proteins in the heart of Tibetan sheep. All sections are from two-year-old sheep. (**A**,**B**): Hypoxia-inducible factor (HIF)-1α immunostaining in the heart; (**C**,**D**): HIF-2α immunostaining in the heart; (**E**,**F**): HIF-3α immunostaining in the heart; (**G**,**H**): Vascular endothelial growth factor (VEGF) immunostaining in the heart; (**I**–**L**): control of HIF-1α, HIF-2α, HIF-3α and VEGF in heart respectively. Arrows in (**B**,**D**,**H**): HIF-1α, HIF-2α and VEGF immunostaining in myocardial nuclei, respectively. The positive immunostaining showed brown with non-immunostained nuclei blue. (**A**,**C**,**E**,**G**,**I**): 20×; (**B**,**D**,**F**,**H**,**J**–**L**): 100×).

**Figure 2:**
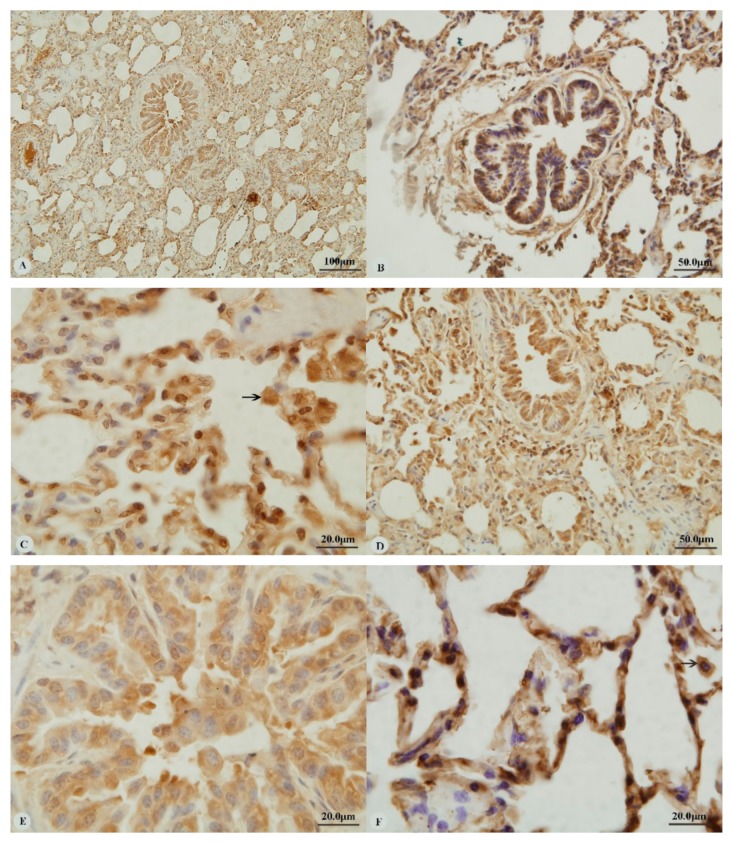

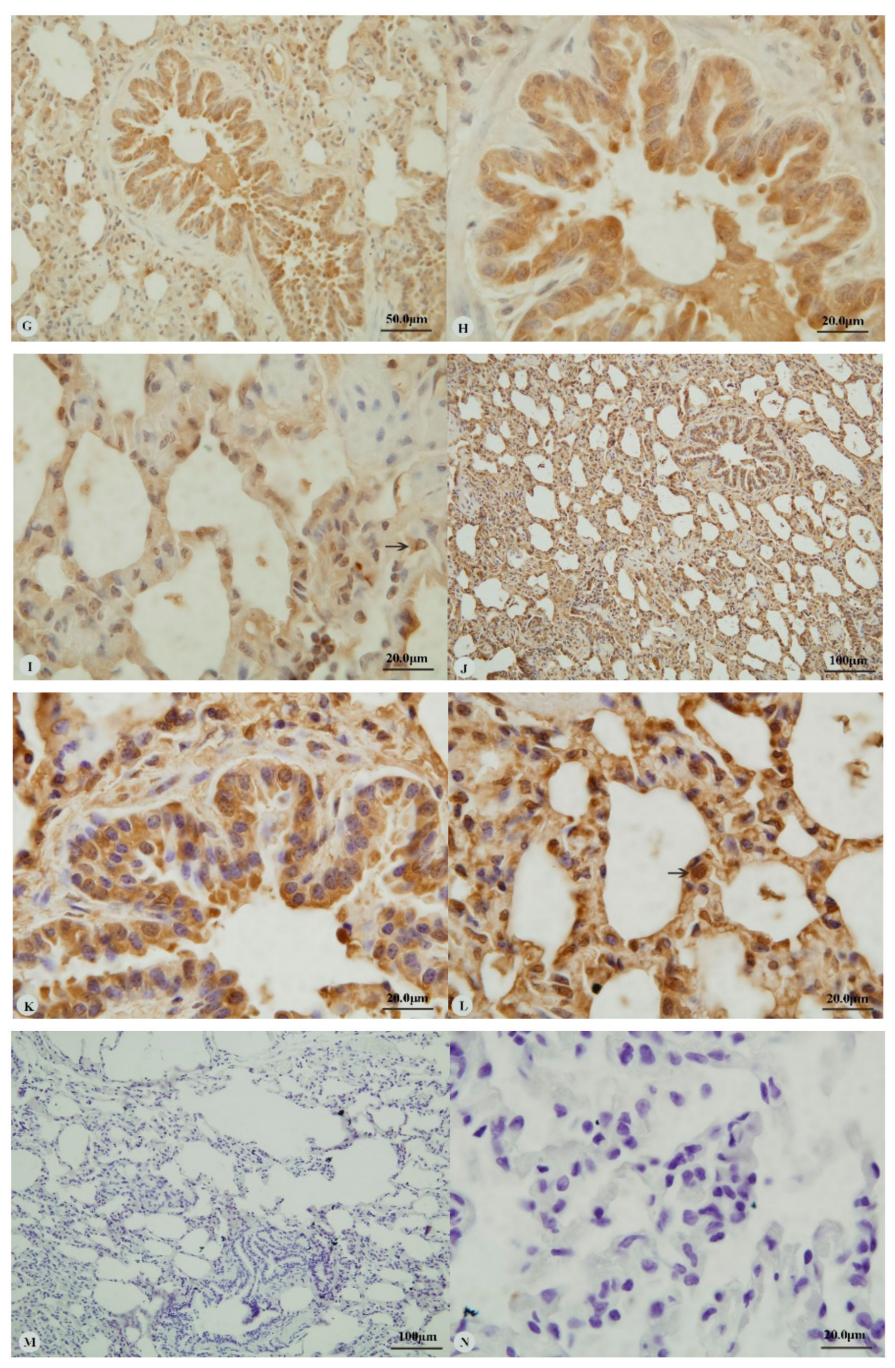

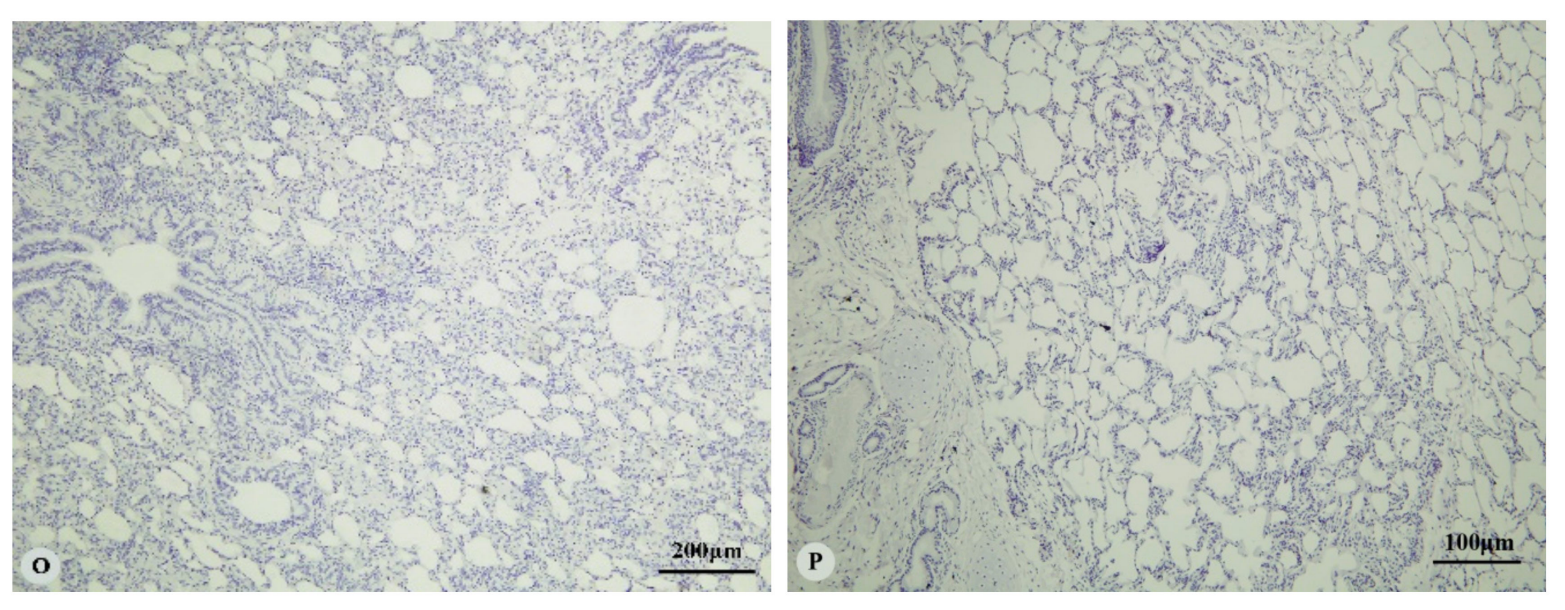
Immunostaining of four proteins in the lung of two-year-old Tibetan sheep. (**A**–**C**): Hypoxia-inducible factor (HIF)-1α immunostaining in the lung; (**D**–**F**): HIF-2α immunostaining in the lung; (**G–I**): HIF-3α immunostaining in the lung; (**J–L**): Vascular endothelial growth factor (VEGF) immunostaining in the lung; (**M–P**): control of HIF-1α, HIF-2α, HIF-3α and VEGF in lung, respectively. Arrows in (**C**,**F**,**I**,**L**): HIF-1α, HIF-2α, HIF-3α and VEGF immunostaining in alveolar macrophages, respectively. The positive immunostaining was brown with non-immunostaining nuclei blue. (**A**,**D**,**G**,**J**,**M**,**O**,**P**): 20×; (**B**,**C**,**E**,**F**,**H**,**I**,**K**,**L**,**N**): 100×).

Examination of the heart revealed immunostaining for HIF-1α, HIF-2α and VEGF in the cytoplasm (Figure 1A,C,E,G) and nucleus (Figure 1B,D,H) of cardiac muscle cells. In contrast, immunostaining for HIF-3α was restricted to the cytoplasm of these cells (Figure 1F). Examination of the lungs (Figure 2A,D,G,J) revealed cytoplasmic immunostaining for all four proteins in the cytoplasm of terminal bronchial epithelial cells (Figure 2B,E,H,K) as well as the cytoplasm and nucleus of pneumocytes (Figure 2C,F,I,L). Cytoplasmic and nuclear immunostaining was also visible within cells interpreted as alveolar macrophages with the immunostaining most intense within the nuclear membrane (Figure 2F,L). Immunostaining varied from stippled and homogeneous in the bronchioles to more punctate and granular in pneumocytes and macrophages. Immunostaining was not observed in the negative control sections (Figure 1I–L; Figure 2M–P). The location of the immunostaining within each tissue was consistent within all three age groups, although as discussed later, there was variability between age groups of the intensity of immunostaining.

**Figure 3:**
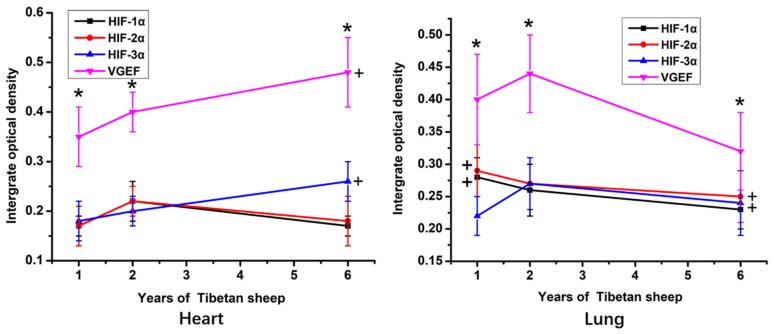
The IOD (Integrated optical density) value of hearts and lungs of Tibetan sheep of different ages. VGEF is vascular endothelial growth factor, HIF is hypoxia-inducible factor. Values are means ± SD (*n* = 10). * indicates that significant differences (*p* < 0.05) in the IOD values existed between immunostaining intensity for VEGF and all three HIFs at that time point. + indicates significant differences in the immunostaining intensity of VEGF and HIF-1α in the heart and HIF-1α HIF-2α in the lung between 6-year-old and 1-year-old sheep.

According to the IOD statistic, within the heart and lung, the immunostaining intensity of VEGF protein was significantly higher than the intensity of immunostaining against the HIFs (*p* < 0.05; Figure 3). The intensity of VEGF and HIF-3α immunostaining in the heart was significantly higher in 6-year-old sheep than in 1-year-old sheep (*p* < 0.05). In contrast, in the lungs, the highest IOD value for VEGF and HIF-3α was in tissue from the 2-year-old sheep. The IOD value of immunostaining against HIF-1α and HIF-2α in sections of lung was significantly higher in the 1-year-old than the 6-year-old sheep (*p* < 0.05).

### 3.2. HIF-1α, HIF-2α, HIF-3α and VEGF ELISA

**Figure 4:**
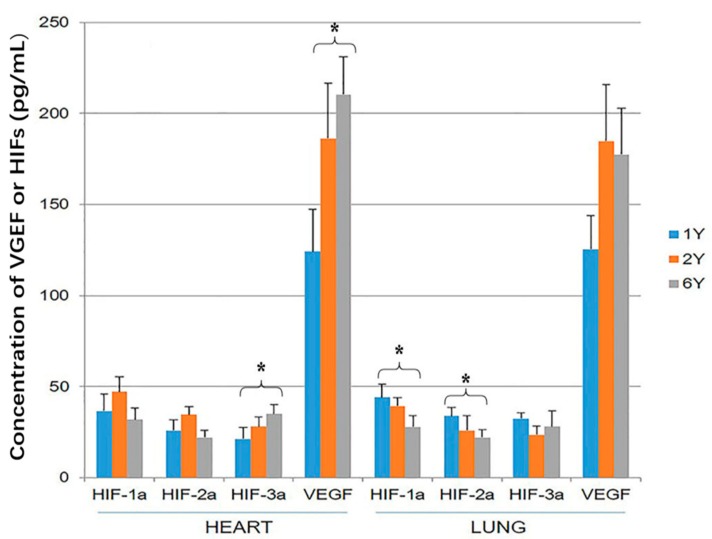
Hypoxia-inducible factor (HIF)-1α, HIF-2α, HIF-3α and vascular endothelial growth factor (VEGF) expression in the heart and lung of Tibetan sheep were measured by ELISA. Values are means ± SD (*n* = 10). * indicates a significant difference in expression between 1-year-old and 6-year-old sheep (*p* < 0.05).

The tissue concentration of VEGF was significantly higher than the concentration of the three HIF proteins within both heart and lung (*p* < 0.05). The concentration of VEGF and HIF-3α was higher in the hearts of 6-year-old than 1-year-old sheep (*p* < 0.05; Figure 4). The concentration of HIF-1α and HIF-2α was higher in the lung of 1-year-old sheep than 6-year-old (*p* < 0.05; Figure 4).

### 3.3. RT-PCR

**Figure 5:**
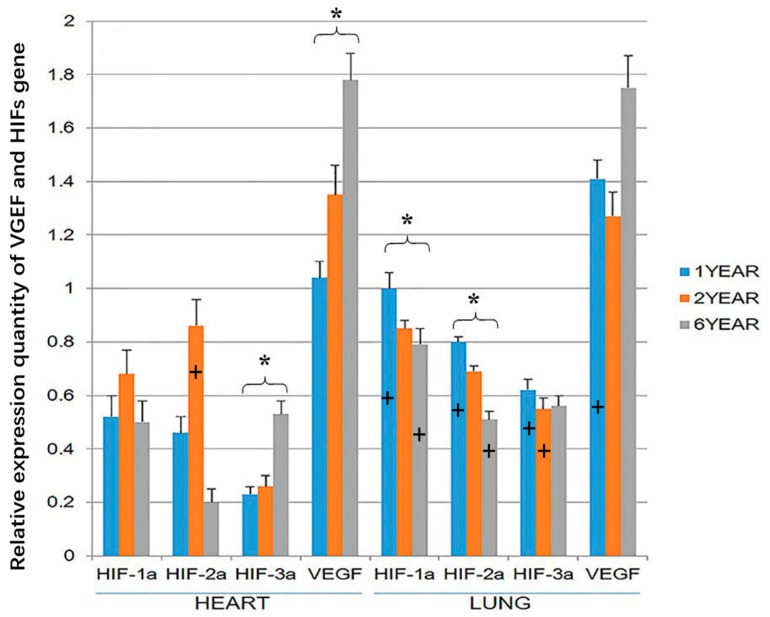
Hypoxia-inducible factor (HIF)-1α, HIF-2α, HIF-3α and vascular endothelial growth factor (VEGF) gene expression in the heart and lung of Tibetan sheep were measured by RTPCR (Reverse transcription polymerase chain reaction). Values are means ± SD (*n* = 10). * indicates a significant difference (*p* < 0.05) in gene expression within a tissue between 1-year-old and 6-year-old sheep. + indicates a significant difference (*p* < 0.05) between gene expression in the heart and the expression of the same gene at the same age in the lung. The bar containing + has the significantly higher gene expression.

VEGF and *HIF-3α* gene expression was higher in the heart of the 6-year-old sheep than the 1-year old sheep (*p* < 0.05; Figure 5). HIF-1α and HIF-2α expression was highest in the hearts of 2-year-old sheep, but this difference was not significant. However, in the lung, HIF-1α and HIF-2α gene expression was higher in the 1-year-old than the 6-year old sheep (*p* < 0.05). At the age of 1 year, the expression of the four genes was significantly higher in the lung than in the heart (*p* < 0.05). The expression of HIF-3a in the lung was higher in the heart of 2-year-old sheep, and at the age of 6-year-old sheep, the expression of HIF-1a and HIF-2a in the lung was higher than in the heart (*p* < 0.05; Figure 5). There were no significant differences in the expression of VEGF in the lung and heart in 2-year-old and 6-year-old sheep (Figure 5). It should be noted that while the gene expression of the housekeeping genes is expected to remain roughly constant within sheep in the three age groups, small changes in GADPH or beta-actin expression could have resulted in changes in the relative expression of VEGF and the HIFs between the three age groups of sheep.

## 4. Discussion

The present study showed widespread expression and immunostaining for proteins that are thought to be involved in adaptation to hypoxia in tissues taken from Tibetan sheep. Expression and immunostaining against these proteins was variable between samples of lung and heart and significant differences in these proteins were identified within sheep of different ages. These results suggest that the HIF proteins and VEGF are important in maintaining homeostasis in Tibetan sheep kept at high altitude and it is possible that these proteins are key in the hypoxia-tolerance that is shown by this species.

While the results suggest that the studied proteins are important in preventing diseases caused by hypoxia in these animals, it would have been interesting to look at the expression and immunostaining of these proteins in Tibetan sheep kept at low altitudes. However, as these sheep are not kept at lower altitudes, this was not possible in this case. While the use of other low-altitude breeds was considered, potential differences in protein expression between breeds could have made such comparisons difficult. Further studies in which Tibetan sheep are deliberately moved from low to high altitudes or high to low altitudes may allow the role of the HIF proteins and VEGF in hypoxia tolerance to be determined.

There has been long-term interest in distinguishing the roles of HIF-1α, HIF-2α and HIF-3α, which in common/contrast with short term reduced oxygen availability, has a broad impact on human and animal physiology, and elaborate adaptive mechanism have evolved in response to hypoxia stress [14,15,16,17]. Although much has been learned about the role of HIF-1α and HIF-2α in activating the transcription of hypoxia-responsive genes, the mechanism by which HIF-3α levels within cells are regulated by hypoxia is still poorly understood. Differences in HIF-1α expression due to age has previously been reported in Yak [13], although differences between Tibetan sheep of different ages have not previously been examined. The present results suggest that increased expression of these proteins develops due to chronic exposure of the animal to hypoxia. In contrast, HIF-3α acts as an inhibitor of HIF-1α and HIF-2α, thus decreasing transcription of these downstream genes. Our studies showed that the expression of HIF-1α and HIF-2α proteins in heart and lung has the same change trend, which suggest the two isoforms are co-expressed. The concentration of HIF-1α and HIF-2α was higher in younger than old sheep suggesting that HIF-1α and HIF-2α may be involved in the initial response of the young to hypoxia. Our results also showed higher expression of HIF-3α and VEGF in the heart of older than younger sheep, suggesting that HIF-3α and VEGF may help prevent damage due to longstanding hypoxia. Our data also supports the suggestion that HIF-3α has a positive relationship with VEGF, but the mechanism of such a relationship remains uncertain. This may suggest that as the chronic hypoxia continues, the body adapts as it ages and the increased HIF3 expression acts to reduce the changes that are induced by HIF-1α and HIF-2α. This supports to previously reported hypothesis that HIF-3α may be important in regulating gene expression in response to hypoxia [17].

Vascular endothelial growth factor has been shown in numerous studies to play a critical role in the development of the cardiopulmonary vasculature and to promote blood vessel development. In the present study, expression of VEGF in the Tibetan sheep was higher than the HIFs in both samples of heart and lung. This may suggest that both HIF-1α and HIF-2α induce VEGF expression and it is possible that a major mechanism by which HIF-1α and HIF-2α protect against hypoxia is by inducing high expression of VEGF. Alternatively, the higher VEGF expression could simply have been due to the presence of amplification mechanisms so that small amounts of the HIF proteins results in a marked increase in VEGF.

Compared with the translated protein of HIF-1α, HIF-2α in lung, HIF-3α and VEGF in the heart of Tibetan sheep, we found that gene expression of the HIF-1α, HIF-2α in lung, and HIF-3α and VEGF genes in heart showed a similar change trend with protein level. It suggests that HIF-1α, HIF-2α protein and gene level decreased during sustained chronic hypoxia in lung, and HIF-3α and VEGF protein and gene level increased following the long-term hypoxia. These observations in agreement with some previous reports [18]. It is also inferred that an inducible expression of these proteins by hypoxia is regulated at the protein level as well as gene levels. On the other hand, in our study, we found that HIF-1α, HIF-2α, HIF-3α and VEGF genes expression were greater in lung than in heart at the 1-year old sheep. One possible explanation for this discrepancy between lung and heart is that, although cardiopulmonary function is a combined organ, the lung epithelium interfaces directly with the atmosphere. Therefore, it is possible that the damage due to hypoxia is greater and earlier in the lung than heart and hypoxia-associated gene expression is initially higher in lung than the heart of Tibetan sheep. It has been previously reported that a high steady-state expression of *HIF-3α* is achieved in alveolar epithelial cells during sustained hypoxia. Additionally, HIF-3α expression was reported in the heart and lung of Tibetan goat and in human pulmonary artery cells during hypoxia [19]. Drevytska et al. [20] also reported HIF-3α expression in the heart, lung, kidney and skeletal muscle of rats in response to hypoxia. In the present study HIF-3α gene expression was greater in the lungs than heart of the 1-year-old and 2-year-old Tibetan sheep, supporting a role of HIF-3α in preventing damage due to hypoxia. We do not agree with the view of Heidbreder et al. [21] who suggests that HIF-3α protects against acute periods of hypoxia with HIF-1α protecting against prolonged hypoxia. Instead, our experiment found that both proteins were expressed over a long period of time in response to hypoxia in these sheep. This may suggest that Tibetan sheep may respond to chronic hypoxia by difference cellular methods that in other species.

Previous research has showed that hypoxia, a decrease in available oxygen reaching the tissue of the body, has profound cellular and metabolic consequences. The cellular response to hypoxia is regulated by the HIF family of proteins [22,23]. It is currently believed that in normal oxygen content conditions, HIF-1α is mainly localized in the cytoplasm. However, under hypoxic conditions, HIF-1α translocates to the nucleus where it dimerizes with HIF-1β, activating downstream target genes that mediate the cell changes that protect against hypoxia [13,24]. Although the activation of HIF-1a is well established, there is currently much less information describing the activation and action of HIF-2α and HIF-3α. In the present study, HIF-1a and 2a immunostaining was cytoplasmic and nuclear while HIF-3α was only cytoplasmic. This suggests that in Tibetan sheep not only HIF-1α protein, but also HIF-2α protein may translocate to the nucleus of cardiac muscle cells in response to hypoxia. As HIF-3α remained restricted to the cytoplasm of these cells, it is uncertain how this protein mediates its effects on cell regulation, but potentially this could be by post-translational regulation of HIF-1α and HIF-1α. In the present study immunostaining for all four proteins was present in the cytoplasm of terminal bronchial epithelial cells as well as the cytoplasm and nucleus of pneumocytes. This distribution is similar to the previously reported distribution of these proteins in the pulmonary vasculature of Yaks [25].

There is evidence that alveolar hypoxia induces lung inflammation with activated alveolar macrophages initiated a systemic inflammation response by releasing circulating pro-inflammatory mediators [26,27,28,29,30]. In our study, we found positive immunostaining for HIF-1α, HIF-2α, HIF-3α, and VEGF within alveolar macrophages. The results add additional evidence that macrophages can alter their gene expression in response to environment changes such as hypoxia. Recent studies have shown an important role of metabolism for macrophage polarization and function [31,32,33]. Macrophages reprogram their metabolism and function according to the environment conditions and stimuli in order to polarize for pro or anti-inflammation cells. We argue that this ubiquitous and obvious feature of adaption to a hypoxic environment constitutes a cytokine-like action and functions to increase the production of hypoxia inducible factors.

The heart and lung play an essential role in physiological homeostasis, especially in the hypoxic environment. In this study, we chose to look at both heart and lung, not only to report the expression of these factors in each tissue, but also to investigate any temporal relationship between them for expression of these factors under a hypoxic environment. The data represents an example of a growing body of evidence regarding systemic effects of alveolar macrophages activation induced by hypoxia. The research could provide insight into the role of inflammation in conditions associated with reduced alveolar PO2, and further understanding of this phenomenon should provide insights into the adaptation of the human heart and lung at high altitudes. This may suggest novel approaches to prevent chronic mountain sickness of Tibetan people who lived in Qinghai-Tibetan plateau.

## 5. Conclusions

The following conclusions can be drawn from this study: (1) Hypoxia can induce the translocation of HIF-1α, HIF-2α and VEGF into the nucleus in the heart. (2) HIF-3α and VEGF played an important role in heart responding to hypoxia damage with HIF-1α and HIF-2α involved in an adaptive response to changes due to hypoxia. (3) Alveolar macrophages play an important role in prevention of tissue damage due to hypoxia. However, as only one species was evaluated in this study, it cannot be certain that other species will response similarly to Tibetan sheep in hypoxic environments.

## Supplementary Materials

The following are available online at https://www.mdpi.com/2076-2615/9/9/673/s1, Figure S1: Primers information. Table S1: The melting curves of *HIF-1α, HIF-2α, HIF-3α, VEGF* genes and the reference gene 18S.
