# Supplementary material for: Association of Age with the Expression of Hypoxia-Inducible Factors HIF-1α, HIF-2α, HIF-3α and VEGF in Lung and Heart of Tibetan Sheep
Source: Animals (Basel). 2019 Sep 11;9(9):673. doi: 10.3390/ani9090673 (PMC6769909; doi:10.3390/ani9090673)
Supplement: Supplementary file 1 [file animals-09-00673-s001.pdf]

animals

MDPI

Supplementary files: Association of age with the expression of hypoxia-inducible factors HIF-1α, HIF-2α, HIF-3α and VEGF in lung and heart of Tibetan sheep

Yanyu He [1], John S Munday [2,*], Matthew Perrott [2], Guan Wang [3] and Xiu Liu [3,*]

Table S1. Primers information.

Primer.	Primer sequence	Length (bp)	Tm/℃
HIF-1α	TTGTGACCATGAGGAAATGAGA<br>TTCCATGTTGCAGATTTTATGTT	394	61.5
HIF-2α	CCTACTGCGACGACAGAATCA<br>GTTCTGGTGGCTTTTGGTCA	98	**60**
HIF-3α	AGAGAACGGAGTGGTGCT<br>ATCAGCCGGAAGAGGACTTT	301	60
VEGF	AAAGCCAGCACATAGGAGAGA<br>CAAATGCTTTCTCCGCTCTGA	194	60
18SrRNA	AGCCTTCCTTCCTGGCATGGA<br>GGACAGCACCGTGTTGGCGTAGA	178	60

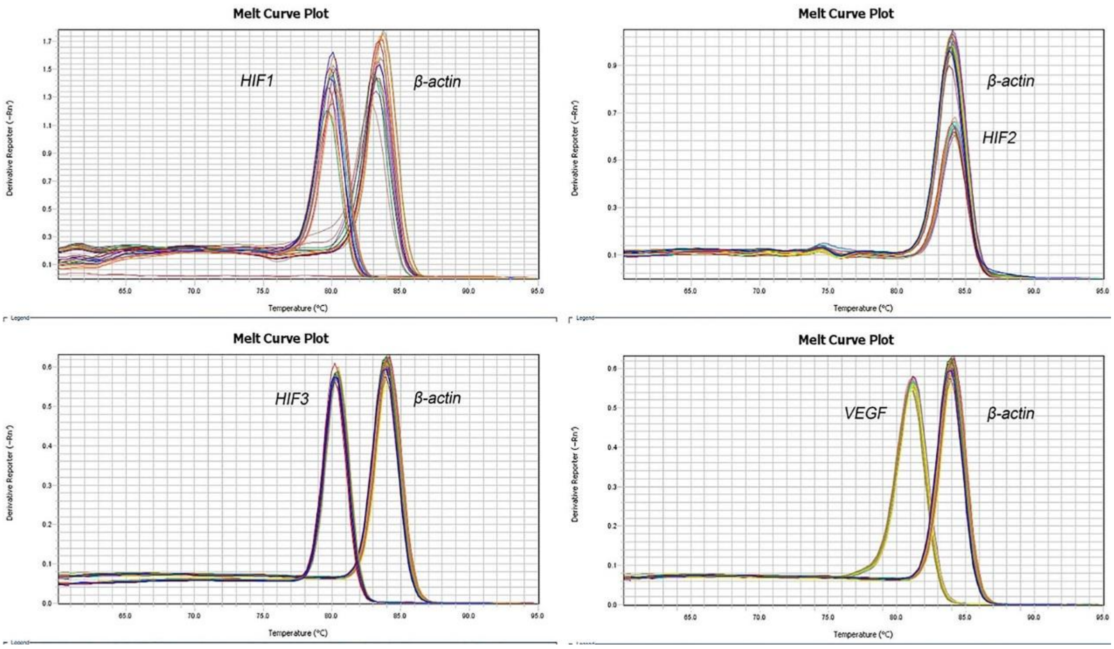

Figure 1. The melting curves *of HIF1α, HIF2α, HIF3α, VEGF* genes and the reference gene 18S.
